# Development and validation of the Physician’s Health Literacy Competence Scale: A step towards effective doctor–patient communication

**DOI:** 10.1097/MD.0000000000041643

**Published:** 2025-02-28

**Authors:** Chia-Chen Chang, Pei-Ling Tseng, Chun-Cheng Liu, Jin-Lain Ming, Su-Hao Fan, Chen-Yin Tung

**Affiliations:** a Department of Health Promotion and Health Education, College of Education, National Taiwan Normal University, Taipei, Taiwan; b Department of Nursing, Taipei Veterans General Hospital, Taipei City, Taiwan; c Department of General Medicine, Cardinal Tien Hospital, New Taipei City, Taiwan.

**Keywords:** health literacy, medical education, physician–patient relationship, scale, shared decision-making

## Abstract

This study aimed to develop a physician’s Health Literacy Competence Scale that emphasizes the importance of enhancing patient health literacy for effective patient-centered care. A literature review of health literacy and existing measurement tools was conducted to develop the scale’s structure and items. The scale was refined through a cross-sectional design in 2 stages: item testing and examination of reliability and validity. Exploratory factor analysis was conducted on the pilot test results of 127 medical students. Items with cross and low factor loadings were deleted, resulting in the retention of 25 items. Subsequently, 203 medical students were recruited as samples for confirmatory factor analysis. Items with excessively large modification indices were excluded from analysis. Finally, 20 items were retained, comprising 4 factors: building doctor–patient trust, providing a supportive environment, shared decision-making, and verifying medication adherence. Analysis of content, internal consistency, and construct validity confirmed the strong reliability and validity of the Physician’s Health Literacy Competence Scale. This effective tool serves curriculum development and assessment in medical education while also offering valuable insights into potential reforms in related courses.

## 1. Introduction

The growing complexity of healthcare, fueled by advancing medical technology, is putting strain on physician–patient relationships, often leading to disputes.^[1–6]^ A patient-centered approach with strong communication skills and health literacy is essential to address these challenges.^[7,8]^ Physicians with health literacy competencies can improve patients’ understanding and adherence, enhancing healthcare quality and relationships.^[9–11]^ Recognizing this, organizations emphasize culturally and demographically tailored communication, while agencies like the US Agency for Healthcare Research and Quality highlight health literacy as key to effective healthcare and strong physician–patient relationships.^[12]^

Medical education emphasizes medical knowledge and clinical skills and overlooks the critical role of patient health literacy in effective healthcare delivery.^[13]^ This knowledge gap has limited physicians’ ability to accurately assess patient needs and deliver patient-centered care.^[7,8]^ Recognizing this limitation, medical institutions have begun integrating health literacy training into their curricula.^[14]^ Existing research provides compelling evidence for the benefits of health literacy training. Studies in 2016 demonstrated that physician training in health literacy and communication skills led to increased engagement in shared decision-making (SDM) with patients, improved patient satisfaction, and enhanced outcomes in hypertension management and medication adherence.^[15]^ A 2020 study by Kaper et al further reinforced the efficacy of health literacy training, reporting significant improvements in medical student competence following an 11-hour program^.[16]^ Despite these promising findings, existing health literacy initiatives often lack a comprehensive framework that encompasses the core components of this competency^.[17]^

Historically, health literacy research has primarily focused on assessing patients’ ability to access, comprehend, and utilize health information.^[17,18]^ This focus stems from the recognition that low health literacy can negatively impact patients’ health outcomes.^[19–21]^ However, a growing body of research highlights the crucial role of physician health literacy in facilitating effective communication and empowering patients in healthcare decision making^.[15,22,23]^ Foundational models, such as Nutbeam levels of health literacy and Sørensen et al’s comprehensive framework,^[24,25]^ highlight health literacy’s role in healthcare quality but focus primarily on general patient literacy rather than specific competencies needed in clinical interactions.

Physician health literacy encompasses a combination of knowledge, attitudes, and communication skills.^[22]^ Knowledge elements include understanding health literacy concepts, cultural influences on health literacy, and the relationship between health literacy and health outcomes.^[26]^ Attitudinal aspects focus on valuing clear communication and providing patient-centered materials.^[23,27]^ Finally, communication skills encompass the ability to tailor medical information to different literacy levels, actively listen to patients’ concerns, and involve them in SDM. Research increasingly emphasizes physician health literacy’s role in communication and patient empowerment.^[23,27]^ This competency involves knowledge (health literacy concepts and cultural influences), attitudes (valuing clear communication), and communication skills (tailoring information, active listening, and SDM).^[27]^ The framework by Liu et al exemplifies this shift, outlining core dimensions such as communication and knowledge.^[23]^ Recognizing the importance of physician health literacy fosters patient-centered care, improved communication, and ultimately better health outcomes.^[7,8]^

This study aimed to develop and validate a reliable and effective assessment tool, the Physician Health Literacy Competence Scale (PHLCS), to evaluate physicians’ professional competence in health literacy. Focus group interviews and expert evaluations were conducted to define the scale’s dimensions, followed by content validity testing to ensure that the assessment items accurately capture the core health literacy skills essential for physicians.

## 2. Materials and methods

This study followed a cross-sectional, three-stage scale development process, inspired in part by the Jandhyala method for assessing proportional group consensus and adapted using neutral theory to minimize bias.^[28]^ The stages included item construction, item testing, and validation of scale reliability and validity. Initial item development was informed by a comprehensive literature review and expert content validity analysis. Subsequently, medical students who had completed clinical rotations were recruited to participate in a pretest and a formal survey. The study utilized Exploratory factor analysis (EFA) (n = 127) and confirmatory factor analysis (CFA) (n = 203) were used to refine the items. The sample size selection was based on established psychometric guidelines, which recommend a minimum of 100 participants for factor analysis.^[29]^ Additionally, Gorsuch suggests that a common rule of thumb for sample size is to have at least 5 times the number of items in the scale to maintain analytical stability.^[30]^ While this guideline is frequently cited in factor analysis research, its application to CFA is contingent upon factors such as model complexity and indicator reliability. In this study, these criteria were systematically applied to strengthen the robustness, validity, and generalizability of the findings.

Ethical approval for this study was obtained from the Institutional Review Board of National Taiwan Normal University (Approval No: 201809HS007). All procedures were conducted in accordance with relevant ethical guidelines and regulations. Informed consent was obtained from all participants prior to their involvement in the study.

### 2.1. Item construction: Initial development of the physician’s competence on health literacy scale

This section details the initial developmental phase of physicians’ competence on a health literacy scale. Following Jandhyala’s framework for generating a consensus-driven item list, a systematic literature review (2010–2020) across CINAHL, Cochrane Library, PubMed/MEDLINE, and CEPS databases informed the item construction process. The research team used thematic coding to translate free-text responses into distinct, mutually exclusive items for the health literacy competence scale.^[23]^. This comprehensive review encompasses studies exploring health literacy from diverse perspectives, including those of the general public, patients, and healthcare professionals.

The findings from the literature review informed the development of this framework, which integrates key concepts from various sources, including health literacy itself, existing literacy assessment tools, factors influencing patient acceptance, shared medical decision-making, and the teaching-back method.^[23,27,31,32]^ Through rigorous analysis and synthesis by the research team, 5 core dimensions were identified.

#### 2.1.1. Concepts and evaluation

This dimension addresses the foundational understanding and value of health literacy, including its various manifestations, particularly those associated with low health literacy, as well as the tools and methodologies used to assess patients’ HL.

#### 2.1.2. Respect and acceptance

This dimension emphasizes the importance of patient-centered care by fostering a climate of acceptance and empathetic understanding.

#### 2.1.3. Communication and interaction

Encompassing nonverbal communication, effective language utilization, and the strategic use of educational materials, this dimension highlights the importance of creating a patient-centered communication environment.

#### 2.1.4. Information use and decision-making

This dimension focuses on ensuring clarity in medical information sharing, facilitating collaborative decision-making processes with patients, and confirming their understanding of medical choices.

#### 2.1.5. Feedback and teaching back

This final dimension emphasizes the importance *of* confirming patient comprehension, implementing the teach-back method for verification, and providing constructive feedback to enhance patient knowledge and engagement.

The framework encompasses 5 main dimensions, further elaborating on 16 subdimensions, with a total of 27 indicators.

The scale items were developed by referencing the literature on health literacy competence among medical providers, particularly medical students and physicians. The survey design was inspired by the Objective Structured Clinical Examination used in clinical teaching in medical schools. Administered to medical students, it included 4 simulated medical interaction scenarios. It covers attitudes with 8 items and skills with 32 items. Responses were rated on a Likert scale ranging from 1 (strongly disagree) to 5 (strongly agree) for attitude items, and from 1 (very uncertain) to 5 (very confident) for skill items, resulting in a 40-item scale.

To establish content validity, 6 experts in healthcare decision-making, health literacy, and medical education, along with a physician specializing in medical ethics and doctor–patient communication, conducted a content validity index assessment. The panel included 4 university faculty members, 1 physician, and 1 government health insurance representative, ensuring diverse expertise. Each expert systematically evaluated the questionnaire for relevance and clarity, providing iterative feedback. Based on their input, the questionnaire was refined before being tested in a pilot study to assess its clarity and feasibility.

### 2.2. Pilot study

A purposive sampling approach was used to administer the pilot scale to participants across 3 medical schools, specifically targeting 5th- and 6th-year medical students in clinical clerkship, postgraduate year residents, postgraduate year 1 residents, and postgraduate physicians in specialty training. Participants rated their level of agreement anonymously, and each item’s awareness and agreement were evaluated to ensure objectivity, following neutral theory principles. Invitations were disseminated through class networks, and data collection was conducted via a web-based survey in July 2019, resulting in 130 responses. After excluding ineligible or incomplete responses, 127 valid responses were obtained, yielding an effective response rate of 97.6%.

### 2.3. Sample

Using purposive sampling, medical students from 2 medical schools were recruited for the main study. In alignment with neutral theory principles, data were collected anonymously to reduce potential response bias and accurately gauge item awareness. A web-based survey was conducted from September to November 2019. Of the 211 contacted participants, 5 declined participation, and 3 provided duplicate responses. The final sample comprised 203 participants, with a response rate of 96.2%. The sample demographics were as follows: 149 males and 54 females, including 97 fifth-year students, 40 sixth-year students, and 30 seventh-year students.

### 2.4. Measures

The scale consisted of 47 items distributed across 4 dimensions, each representing key aspects of clinical doctor–patient interaction scenarios. A content validity index analysis was conducted to assess the relevance, importance, and clarity of each item, using an average score approach. Results indicated that the “Concepts and Assessment” dimension had a content validity index of 0.78, while all other dimensions scored above 0.80. Based on these findings, all items were retained, though minor revisions were made to certain items’ wording following expert feedback. The final pilot scale consisted of 47 items, with 40 items retained for statistical analysis after excluding 7 knowledge-based items from the scale analysis.

Following the scale administration, EFA was conducted using SPSS for Windows version 23.0 (n = 127) to examine construct validity. CFA was subsequently performed using Mplus version 8.2 (n = 203) to evaluate the scale’s structural validity, as well as its convergent and discriminant validity.

### 2.5. Statistical analysis

After collecting the pilot-scale data, data coding and entry were performed and invalid scales were screened using SPSS for Windows version 23.0. Item analysis and internal consistency reliability testing were conducted for the scale.

For content validity, experts assessed item appropriateness and format. Items with extreme average scores (>4.50) or low standard deviation (<0.66) were excluded. Internal consistency reliability was assessed using Cronbach α coefficient, aiming for values between 0.80 and 0.90.^[33]^ EFA was conducted after assessing data suitability using the Kaiser-Meyer-Olkin and Bartlett tests. Principal components analysis with Varimax rotation extracted factors with eigenvalues > 1, considering factor loadings > 0.40.^[34]^ CFA employs fit and modification indices for model modification.^[35]^ Standardized factor loadings, composite reliability, and average variance extracted (AVE) were used to assess convergent validity. Discriminant validity was confirmed by smaller interconstruct correlations.

**Figure 1.**
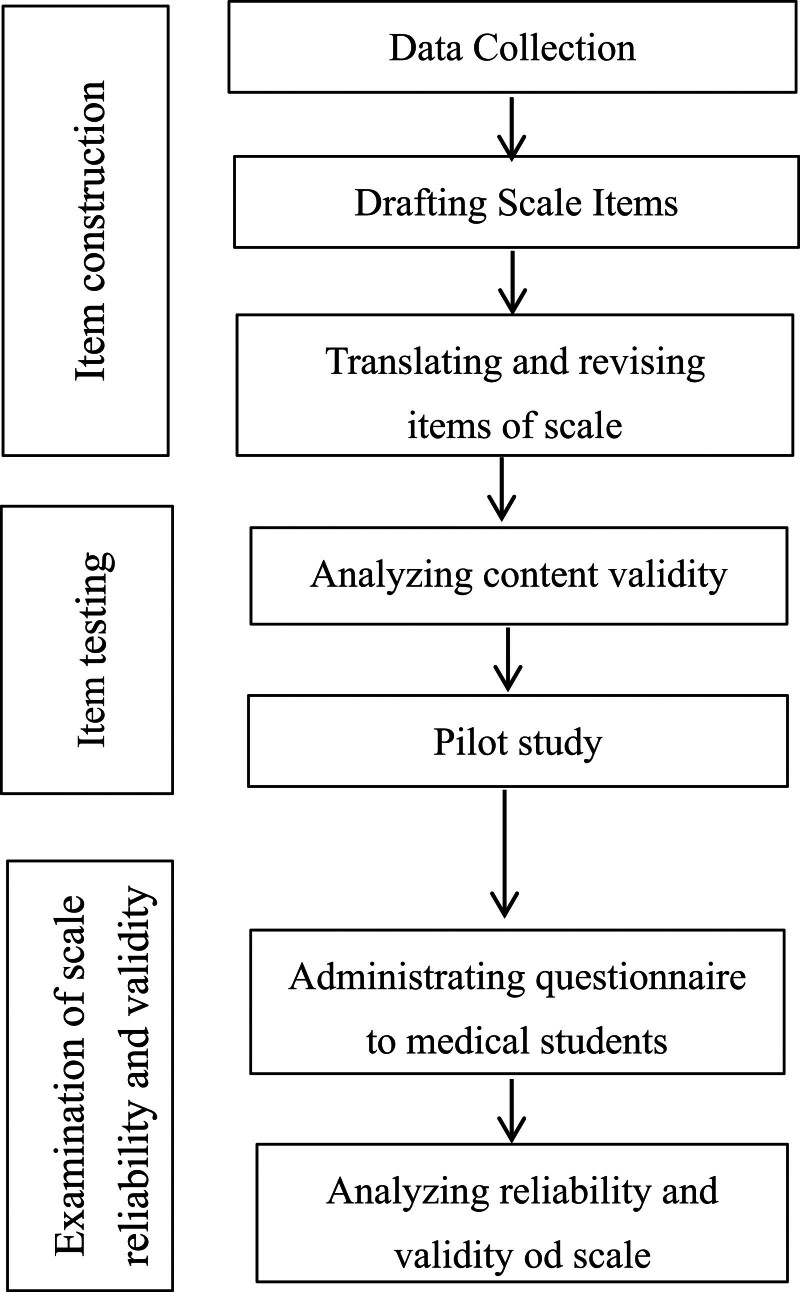
Development process of the Physician’s Health Literacy Competence Scale.

A factor analysis was used to confirm the scale’s structure, followed by internal consistency reliability testing (Cronbach α). Validity tests examined factorial, convergent, and discriminant validity, which informed the development of the Physicians’ Health Literacy Competence Scale framework illustrated in Figure 1.

## 3. Results

### 3.1. Item analysis

Item analysis and internal consistency of the pilot scale were analyzed. An independent sample *t*-test was conducted to examine whether there were differences between the high- and low-score groups. All items in each dimension passed the test, indicating good discriminative ability. The Cronbach alpha reliability coefficient for the scale was 0.944, indicating excellent internal consistency and the ability to distinguish health literacy among medical students. Therefore, 40 items with discriminative ability were selected as the basis for the EFA of the formal scale.

### 3.2. Factorial validity and internal consistency reliability

The results of the CFA fit test showed a Kaiser-Meyer-Olkin measure of sampling adequacy of 0.861 (>0.60) and a statistically significant Bartlett test of sphericity (*P* < .001), indicating that the variables had common factors and were suitable for factor analysis. The factors were extracted individually and then deleted and reviewed according to the 4 factors set in the scale development process. The scree plot and factor-loading results for each item were carefully examined. The results showed that the distribution of each item was consistent with the theoretical structure, with a cumulative variance of 60.87%.

Item analysis was conducted on 40 items. 15 items were deleted in this stage, including 14 cross-factor items that did not fall into a single corresponding factor, and 1 item with a factor loading <0.4. The remaining 25 items were used to analyze the indicators of the 4 factors of medical student health literacy according to the results of the indicator analysis. The attributes of the indicators were summarized, and the 4 factors were named F1 “Shared Medical Decision Making” (9 items), F2 “Confirmation of Medical Order Execution” (6 items), F3 “Building Doctor–Patient Trust” (5 items), and F4 “Providing a Friendly Environment” (5 items). The indicators for each factor were ranked according to their weights. The factor loading results for each item after orthogonal rotation are listed in Table 1.

**Table 1:** Summary of exploratory factor analysis results (n = 127).

Item	Factor loading			
	F1	F2	F3	F4
1. I will analyze the costs, risks, prognosis, and recovery processes of various stent options for patients, allowing them to make informed choices and be willing to accept the consequences of their decisions.	.740			
2. I can clearly explain the purpose of stent placement and the possible consequences of not using a stent, encouraging them to participate in the discussion.	.693			
3. Based on the patient’s clinical symptoms, I can specifically and clearly explain the purpose of the treatment or the possible future progression of the condition.	.691			
4. I can provide patients with easy-to-read pamphlets or manuals on cardiac catheterization surgery for their use at home.	.689			
5. I will allow patients to fully express their treatment preferences, respect their decisions, and proceed only after obtaining their signed consent.	.665			
6. I will discuss the department to which the patient will be referred, the specialist’s expertise, and the consultation situation with the patient and proceed with the referral only after obtaining their consent.	.543			
7. I can proactively clarify patients’ misconceptions about “using a needle to prick a finger to bleed as a treatment during a heart attack.”	.542			
8. I will use questioning techniques to guide patients to express their real medical issues and conditions.	.518			
9. I will ask patients who suddenly enter the consultation room to wait outside to protect the privacy of the previous patient.	.416			
10. I will guide patients in practicing eye drop administration and provide feedback to ensure the correct method is used.		.785		
11. I will choose the appropriate time to have patients repeat the medication administration process in their own words to ensure complete understanding.		.763		
12. I can assess the correctness of the patient’s eye drop administration technique and steps, and clarify or supplement any incorrect parts.		.737		
13. I can timely express recognition or appreciation when the patient correctly performs the eye drop administration technique.		.721		
14. I can slow down my speaking rate and use an appropriate volume to communicate with patients.		.671		
15. I can patiently discuss potential tests that may need to be done, focusing on issues of preference or concern to the patient.		.606		
16. I can arrange a comfortable environment for communication with patients.			.778	
17. I believe that a physician’s ability to grasp a patient’s capability to obtain, understand, and apply medical information is helpful in establishing a good doctor–patient relationship.			.754	
18. I will use an appropriate doctor–patient interaction model based on the patient’s health literacy.			.748	
19. I believe that physicians should empathize with and accept the anxiety patients feel about myocardial infarction.			.735	
20. I think physicians should invite the patient’s family members into the consultation room to provide her with more support and confidence.			.645	
21. During consultations, I will first verify the patient’s medical history, lifestyle habits, or economic status.				.765
22. I can use or seek assistance to explain the medical condition in Minnan, a language familiar to the patient.				.645
23. I believe that the consulting physician should listen patiently to the patient’s statements without interrupting.				.641
24. I will ensure to provide patients with appropriate medical information, not exceeding 3 new concepts.				.539
25. I can create a relaxed atmosphere that makes patients feel that the doctor is approachable.				.473

**Figure 2.**
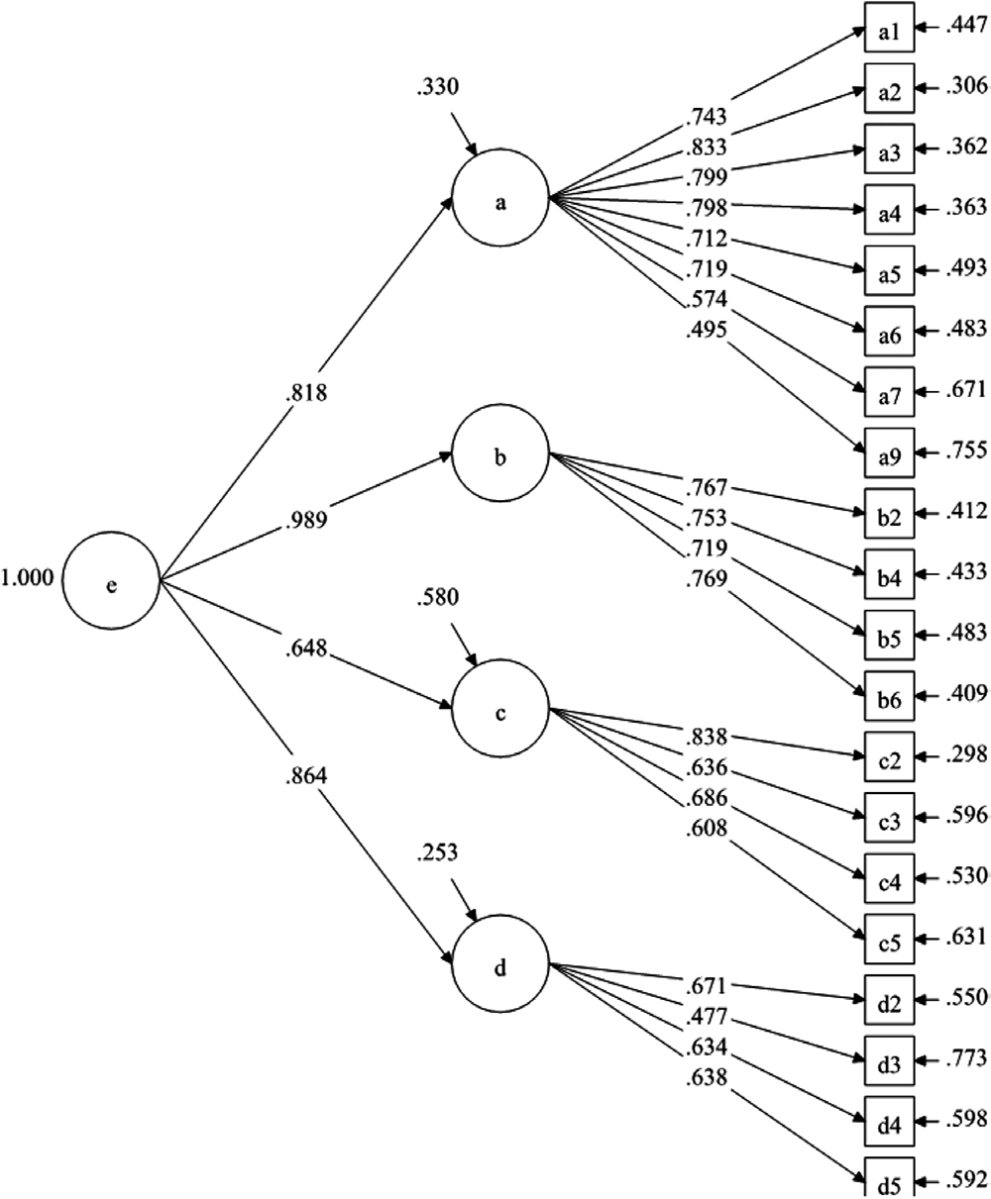
Final model of the Physicians’ Health Literacy Competence Scale.

The skewness and kurtosis coefficients of all variables in the sample were <2 and 3, respectively, indicating that the data were normally distributed. These results are detailed in Table 2. The model fit of the factor structure was further examined. A first-order orthogonal factor model was formed by setting the maximum variance between factors in the model setting. According to the literature, high-order factors affecting respondents’ health literacy include patient health literacy awareness and assessment, communication and interaction, medical information and decision-making, and communication environment.^[31,32]^ Therefore, second-order CFA was conducted to test the model, as presented in Figure 2.

**Table 2:** Summary of fit indices for measurement models.

Construct	χ^2^	χ^2^/dƒ	RMSEA	SRMR	CFI	TLI
Initial One-Factor Model (25 items)	661.50†	2.46*	.09	.08	.85	.84
Initial Two-Factor Model (25 items)	664.83†	2.45*	.09	.08	.85	.84
Revised One-Factor Model (20 items)	361.83†	2.21*	.08‡	.06*	.90*	.88
Revised Two-Factor Model (20 items)	363.92†	2.19*	.08‡	.06*	.90*	.88

RMSEA: Measures model error; <0.05 is good, 0.05–0.08 acceptable, >0.10 poor; CFI: Compares model to null model; >0.90 is acceptable, >0.95 good; SRMR: Shows discrepancy between predicted and observed; <0.08 acceptable, <0.05 good; TLI: Adjusts for model complexity; >0.90 is acceptable, >0.95 good.

* indicates a reasonable range but does not meet the standard values (3 < X^2^/df < 5; .05 < RMSEA < .08).

† P < .001.

‡ indicates compliance with standard values (1 < X^2^/df < 3; RMSEA < .05; SRMR < .08; CFI > .90; TLI > .90).

CFI = comparative fit index, RMSEA = root mean square error of approximation, SRMR = standardized root mean square residual, TLI = Tucker-Lewis index.

The initial one-factor and two-factor models, which included 25 items, showed suboptimal fit indices (root mean square error of approximation [RMSEA] = 0.09, standardized root mean square residual [SRMR] = 0.08, comparative fit index [CFI] = 0.85, Tucker-Lewis index = 0.84), indicating a need for further refinement. To improve model fit, 5 items with high modification indices were removed, resulting in a revised model with 20 items. The revised one-factor and two-factor models demonstrated significant improvements, with RMSEA reduced to 0.08, SRMR to 0.06, and CFI increased to 0.90, reaching acceptable standards.^[36]^ These improvements indicate that the revised model has a more desirable statistical fit, with the CFI notably meeting the threshold of 0.90.

Although the chi-square test (χ²) remained significant for both the initial and revised models (e.g., χ²/df = 2.21 for the revised one-factor model and χ²/df = 2.19 for the revised two-factor model), this may be due to the chi-square test’s sensitivity to sample size (Bentler & Bonett, 1980).^[37]^ Therefore, multiple fit indices, including RMSEA, SRMR, and CFI, were considered in assessing model adequacy rather than relying solely on the chi-square test. The improvements in these indices suggest that the revised model is more robust and stable, enhancing its validity and consistency in measuring the 4 dimensions of health literacy.

### 3.3. Convergent and discriminant validity

The revised second- and first-order four-factor models demonstrated similar model fit indices, indicating a good fit with the observed data. This suggests that the 4 factors are correlated, and can be regarded as a single latent higher-order factor, which can be named “ Physicians’ Health Literacy Competence Scale” Based on the results of the revised second-order CFA, the final factor analysis model structure diagram shows that all the observed variables fall within the standard range.^[38]^ The maximum standardized factor loading, λ, in absolute value did not exceed 0.95 (ranging from 0.48 to 0.84). The standard errors of the factor loadings were not too large (ranging from 0.03 to 0.06). The error variances were all positive and significant, indicating that the second-order structural equation model with 20 items did not violate the estimation (see Table 3). The related data are detailed in Table 3.

**Table 3:** Summary of model parameter estimates, composite reliability, and average variance extracted.

Construct	Item	λ	SMC	SE	t	CR	AVE
Factor 1	a1 a2 a3 a4 a5 a6 a7 a9	.74 .83 .80 .80 .71 .72 .57 .50	.55 .69 .64 .64 .51 .52 .33 .25	.04 .03 .03 .03 .04 .04 .05 .06	21.21*** 32.45*** 27.26*** 27.03*** 18.70*** 19.14*** 11.31*** 8.79***	.89	.51
Factor 2	b2	.77	.59	.04	21.83***	.84	.57
	b4	.75	.57	.04	20.97***		
	b5	.72	.52	.04	18.34***		
	b6	.77	.59	.03	22.32***		
Factor 3	c2	.84	.70	.04	22.64***	.79	.49
	c3	.64	.40	.05	12.72***		
	c4	.69	.47	.05	14.70***		
	c5	.61	.37	.05	11.58***		
Factor 4	d2	.67	.45	.05	12.96***	.70	.37
	d3	.48	.23	.06	7.52***		
	d4	.63	.40	.05	12.12***		
	d5	.64	.41	.05	11.89***		

λ: Standardized factor loading; AVE = Average variance extracted; CR = Composite reliability; SE = Standard error of the factor loading; SMC = Squared multiple correlation.

*** *P* < .001.

As shown in Table 4, the standardized factor loadings for all constructs were significant (*t*-tests), with composite reliability ranging from 0.70 to 0.89 and AVE ranging from 0.37 to 0.57. Although the AVE values for F3 and F4 were below 0.50, previous research has suggested that constructs with AVE values below 0.50, but Composite reliability values above 0.60, still possess adequate convergent validity (Fornell & Larcker, 1981).^[39]^

**Table 4:** Summary of correlations between the scale and each factor and discriminant validity.

Construct	Whole scale	Factor 1	Factor 2	Factor 4	Factor 4
Factor 1	.90***	(.71)			
Factor 2	.88***	.71***	(.75)		
Factor 3	.71***	.52***	.56***	(.70)	
Factor 4	.80***	.59***	.67***	.42***	(.61)

Values in parentheses on the diagonal are the square roots of the average variance extracted.

*** *P* < .001.

In terms of discriminant validity, the correlations between the overall scale and the individual scales ranged from 0.71 (high correlation) to 0.90 (high correlation). The correlations between each pair of subscales were all significant and moderate to high, ranging from 0.42 to 0.71. The square root of the AVE of each subscale was greater than all the correlation coefficients between the subscales, indicating that all subscales had good discriminant validity and could measure the 4 distinct latent constructs.

## 4. Discussion

This study developed the PHLCS to enhance patient-centered care by improving key health literacy skills in clinical settings. The PHLCS incorporates dimensions critical to physician–patient interactions, such as trust-building, supportive environments, SDM, and ensuring adherence to medical guidance. The scale extends existing health literacy frameworks, aligning with Nutbeam model, which emphasizes functional skills (e.g., clear communication), communicative skills (e.g., SDM), and critical skills (e.g., patient empowerment), while also addressing the broader societal and interactive contexts highlighted by Sørensen et al,^[24,25]^ viewing health literacy as a multi-dimensional competency.

Healthcare providers play a pivotal role in promoting health literacy by fostering an environment that enables patients to understand, process, and make informed health decisions.^[40]^ This entails using tailored communication strategies that minimize medical jargon,^[41]^ providing visual aids to enhance comprehension,^[42]^ and being culturally sensitive to diverse patient backgrounds.^[43]^ Employing metaphors and mindful body language further strengthens patient-provider rapport, facilitating more effective patient engagement in care.^[44]^ Foundational to patient-centered care are strategies such as active listening, respecting patient perspectives, and recognizing health literacy limitations, which collectively contribute to more efficient healthcare utilization.^[45]^ These practices help bridge communication gaps, enabling patients to better understand their health conditions and available options.

Ensuring adherence to medical directives post-discharge is critical for patient recovery and the overall quality of healthcare.^[18]^ Effective teach-back sessions allow healthcare providers to confirm patients’ understanding of essential instructions, such as medication adherence, rehabilitation protocols, and wound care.^[18,27,46]^ This proactive approach not only supports quicker recovery but also minimizes potential risks associated with misunderstanding discharge instructions.^[15,47–49]^ It underscores the importance of validating patient comprehension to ensure lasting health outcomes.

The scale is grounded in a four-dimensional framework centered on physician–patient communication, resource utilization, and SDM, aligning with key healthcare quality goals and educational competencies.^[50]^ While existing models, such as Nutbeam and Sørensen et al’s frameworks, focus on patient health literacy, they do not assess physicians’ roles in addressing these challenges.^[24,25]^ The PHLCS extends these models by evaluating physicians’ competencies in fostering trust, supporting SDM, and ensuring treatment adherence. Unlike Coleman et al’s educational framework, which emphasizes training,^[29]^ the PHLCS functions as an assessment tool for real-world application. By addressing critical clinical scenarios, it equips future physicians with essential skills to enhance patient-centered care.

## 5. Conclusion

This study presents a novel health literacy scale specifically designed for physicians. Unlike many existing scales that rely solely on the EFA, this study employed a more rigorous approach. CFA was used to evaluate the goodness-of-fit of the proposed four-dimensional factor structure: building doctor–patient trust, creating a supportive environment, SDM, and verifying medication adherence. The results demonstrated excellent psychometric properties of the tool, including high internal consistency reliability, content validity, convergent validity, and discriminant validity. This signifies the effectiveness of the tool in measuring crucial health literacy competencies among physicians.

This scale offers valuable insights into physicians’ performance in key areas that directly affect patient care. The integration of PHLCS into medical education can support competency-based training by assessing students’ health literacy skills at different stages, such as preclinical coursework, clinical clerkships, and postgraduate training. Additionally, the scale can be applied in Objective Structured Clinical Examinations to evaluate medical students’ communication strategies in simulated patient encounters. Furthermore, PHLCS-informed workshops for residents and practicing physicians can help improve SDM and patient-centered communication. Through ongoing instructional assessments using this tool, medical schools can progressively enhance their students’ health literacy competencies, ultimately leading to improved physician–patient interactions and healthcare outcomes.

### 5.1. Limitations

The study’s sample, primarily drawn from Taiwan, may limit the generalizability of the factor structure confirmed through CFA, as regional cultural and healthcare system differences could influence health literacy competencies. Additionally, the small sample size may affect the stability of the model fit and its applicability to broader populations. Cultural factors, such as variations in physician–patient communication norms and differing expectations of health literacy competencies across healthcare systems, may also impact the scale’s effectiveness in other contexts.

Furthermore, the cross-sectional design restricts causal inferences and does not capture how health literacy competencies evolve over time. Future research should address these limitations by employing larger, more diverse, and internationally representative samples, examining cultural influences on health literacy, and incorporating longitudinal studies to track competency development and validate the scale across different settings.

## Author contributions

**Formal analysis:** Chia-Chen Chang, Pei-Ling Tseng.

**Methodology:** Chia-Chen Chang, Pei-Ling Tseng.

**Project administration:** Chun-Cheng Liu, Jin-Lain Ming.

**Resources:** Chun-Cheng Liu, Su-Hao Fan.

**Supervision:** Chen-Yin Tung.

**Validation:** Chia-Chen Chang.
